# Correction: Zhan et al. Study on the Tea Pest Classification Model Using a Convolutional and Embedded Iterative Region of Interest Encoding Transformer. *Biology* 2023, *12*, 1017

**DOI:** 10.3390/biology14111517

**Published:** 2025-10-30

**Authors:** Baishao Zhan, Ming Li, Wei Luo, Peng Li, Xiaoli Li, Hailiang Zhang

**Affiliations:** 1 College of Electrical and Automation Engineering, East China Jiaotong University, Nanchang 330013, China; 2 College of Biosystems Engineering and Food Science, Zhejiang University, 866 Yuhangtang Road, Hangzhou 310058, China

In the original publication [1], concerns were raised about the integrity of the peer-review process; as such, the Editorial Office has conducted an investigation of this article. This process was supervised by a Member of the Editorial Board to ensure full compliance with MDPI’s Editorial Process (https://www.mdpi.com/editorial_process).

As a result of this investigation, the Editorial Board and the authors have agreed to update the aspects listed below.

## 1. Review Reports

The original Reviewer Report No. 3 was removed from the peer review record.

## 2. References

1. The original References “30” and “31” were removed as these were not sufficiently relevant to the study. The citation and reference numbers were also updated accordingly.

- “Le, N.Q.; Nguyen, T.T.; Ou, Y.Y. Identifying the molecular functions of electron transport proteins using radial basis function networks and biochemical properties. *J. Mol. Graph. Model.* **2017**, *73*, 166–178.”
- “Lam, L.H.T.; Do, D.T.; Diep, D.T.N.; Nguyet, D.L.N.; Truong, Q.D.; Tri, T.T.; Thanh, H.N.; Le, N.Q.K. Molecular subtype classification of low-grade gliomas using magnetic resonance imaging-based radiomics and machine learning. *NMR Biomed.* **2022**, *35*, e4792.”

2. The correct reference numbers were added to the citations of “EfficientNet”, “ShuffleNet”, “MobileNets” and “VggNet” throughout the paper.

The authors have fully cooperated with the Editorial Office both during the peer-review process as well as the investigation. These modifications do not affect the results of the paper.

This correction was approved by the Academic Editor. The original publication has also been updated.
